# Charity or Business?

**Published:** 1896-02-08

**Authors:** 


### Editor's Letter-Box.

### CHARITY OR BUSINESS?

Mr. J. S. Wood, the editor of the *Gentlewoman*, writes: My attention has just been drawn to an editorial note in your paper commenting on the gift to hospitals of £200 which we have recently distributed, and stating that if each of the 17,416 voters expended sixpence on the *Gentlewoman* the proprietors have made an "apparent profit of upwards of £230" out of their donations. You are perfectly aware that this is not true; but lest your readers should be deceived by your statement, I beg to point out that those who voted for the charities were our regular weekly readers, who would have bought the paper whether we had given the £200 or not; and, further, as you know, we get fourpence-halfpenny for the paper, and not sixpence. Your arithmetic is bad enough, but the meanness of your suggestion is worse, and comes with ill-grace from a person who poses as a supporter of institutions. It is your duty, if you don't know it, to commend, not to cavil at those who give charitable donations.

January 30th, 1896.

[It is evidently true that the competition in the circumstances stated yielded an apparent profit of £230. The question of the actual profit we did not and do not enter into, but if we are to accept Mr. Wood's assurance that those who voted would have bought the paper whether he had given the £200 or not, it would have seemed a simple plan for him to have given the money outright. This competition was no doubt good business, but we fail to see where charity enters into the tranaction.—Ed. *T. H.*]

